# The W.H.E.E.L.S. Preschool Vision Screening Program's Initial Outcomes for 12,402 Children Screened Using the Plusoptix Photoscreener

**DOI:** 10.1155/2014/793546

**Published:** 2014-07-01

**Authors:** Natario L. Couser

**Affiliations:** Prevent Blindness Mid-Atlantic W.H.E.E.L.S. Program, 11618 Busy Street, Richmond, VA 23236, USA

## Abstract

*Objective*. To report the results of the W.H.E.E.L.S. Prevent Blindness Mid-Atlantic vision screening program that targets preschoolers using the Plusoptix Photoscreener (Plusoptix Inc., Nuremburg, Germany). *Methods*. Trained program staff members conducted vision screenings at up to 113 preschool programs in the Richmond metropolitan area for four consecutive years; a cross-sectional analysis was performed. *Results and Discussion*. From September 2010 to March 2014, 15,075 preschoolers have been offered a free vision screening; 12,402 (82%) have been screened. A total of 3,018 (24%) have failed the screening and were recommended to follow up with an eye care specialist for a comprehensive examination; only 30% reported complying. Significant refractive errors were more frequently the cause for a failed screening. *Conclusions*. The W.H.E.E.L.S. program has identified a high number of preschoolers with significant amblyopic risk factors that were previously unknown to be present. Undesirably low follow-up reporting outcomes from children who fail a vision screening examination were consistent with other reports. Nevertheless, having a mobile vision screening program that uses photoscreening technology in targeting children prior to school entry is an efficient and cost-effective way to detect vision disorders in a timely manner.

## 1. Introduction

Maintaining healthy vision plays a critical role in a child's overall success at school. Early detection of vision problems in children is essential, as failure to identify and implement an effective treatment in a time sensitive manner can result in a permanent reduction in visual acuity that could have been otherwise prevented [1]. Comprehensive eye examinations that include a complete and detailed inspection of the visual pathway and eye anatomy are indispensable for individuals with known or suspected problems, risk factors, or positive family history of eye disease. However, in comparison, routine pediatric vision screenings are cost-effective and practical and can be a highly accurate means of quickly identifying children that should be referred to have a more detailed examination by an eye care specialist trained at managing pediatric ophthalmic conditions [2, 3].

The W.H.E.E.L.S. (Where Healthy Eyes and Ears Lead to Success) Kindergarten Readiness Program is a mobile vision and hearing screening initiative for preschools in the Greater Richmond, Virginia Area. The program was created and is sustained by the Medarva Healthcare Foundation and Prevent Blindness Mid-Atlantic, is offered at no cost to the children or schools involved, and specifically targets 4-year-old preschool programs. This target age allows for an effective treatment strategy to be implemented if necessary prior to school entry [3, 4]. Amblyopia, defined as a decreased vision caused by poorly developed visual areas of the brain from inadequate early visual experience, occurs in 1% to 4% of children; risk factors for this eye disorder can be detected in a matter of seconds with a high accuracy during a vision screening examination using photoscreening technology [5–7]. In the United States, there is a high variability in vision screening recommendations since each state mandates their own policy. Furthermore, there are currently a paucity of reports presenting information from a large-scale mobile vision screening program using photoscreening technology that targets 4-year-olds. My objective is to assess and present the vision screening outcomes of the W.H.E.E.L.S. program that have been collected for the past four years of the program's existence, with an emphasis on the most current screening year conducted.

## 2. Patients and Methods

The W.H.E.E.L.S. program began delivering vision screenings in 2010 using the Plusoptix S09 Vision Screener (Plusoptix Inc., Nuremburg, Germany). In 2013 for year 4 of the screening program, the S09 model was replaced with the newer Plusoptix S12 Hand-held Photoscreener (Plusoptix Inc., Nuremburg, Germany). There is also a hearing screening component of the program that utilizes the Welch Allyn OAE Hearing Screener (Welch Allyn Inc., Skaneateles Falls, NY) and an educational component provided for the children, parents, and teachers; the related diagnostic outcomes of the hearing screening and the details of the educational curriculum will be addressed in a later manuscript. Trained and credentialed W.H.E.E.L.S. program staff members conduct the noninvasive screenings 4 days per week, for 9 months each year. The W.H.E.E.L.S. program currently serves 113 preschool programs. The population of the children represented varies from Head Start programs, Virginia Preschool Initiative programs, child day care centers, and public schools and private preschools within the Richmond metropolitan area. Prior to each screening visit permission is obtained from the parents or guardian, and directly after the completion of the screening a printed certificate showing the screening results and a letter of explanation are provided to the parents, as well as referral documentation recommending that the child seek care from a pediatric eye doctor if the child does not pass the screening. Referrals are not made to specific doctors or specialists. All data obtained is deidentified to maintain confidentiality. The diagnostic measures for the children screened are as follows: anisometropia, astigmatism, myopia, hyperopia, anisocoria, and gaze asymmetry. Pupillary distance is also documented. See Table 1 for the referral parameters used for the children screened.

## 3. Results and Discussion

The W.H.E.E.L.S. program was initiated in September of 2010 and has provided vision screenings in the Greater Richmond, Virginia area, each of the four years of the program. As of March of 2014, 15,075 preschoolers have been offered a free vision screening from 50 public school locations and 63 private pre-K programs. A total of 12,402 children have received a vision screening by the program, which represents 82% of screenings offered; see Table 2. The remaining 18% of children that did not receive a screening after the offer and consent were sent to the parents were either opt-outs, absent during the screening visit, or due to no response from the parent giving permission for the screening to take place. A total of 3,018 children, or 24% of children screened throughout the four years, have had an abnormal parameter on the screening. Parents receive a screening certificate reporting the results of their child's vision screening rather pass or fail, which also contains information about the screening process and the importance of healthy vision in children especially as it pertains to their performance in the classroom. Additionally, the parents or guardians of the children that represented a failed exam were subsequently provided with referral documentation recommending that the child seek care from a pediatric eye care specialist. All caretakers are also provided with a postcard mailing requesting self-reporting as to whether their child has received follow-up care as a result of their screening. The percentage of children that reported receiving a comprehensive eye examination after failing a screening exam from the program is 30%, even with aggressive reporting measures in place that includes preschool director reporting, returned referrals from medical professionals, and parent self-reporting on returned postcards as previously noted. Poor reported follow-up rates following failed vision screenings have been previously recognized [8, 9]. It is possible that a higher percentage of children referred due to a failed vision screening followed up appropriately with an eye care specialist without reporting the follow-up visit. Nevertheless, low reported follow-up rates raise concerns that the child referred is at risk for not receiving the appropriate care in a timely manner and also create limitations in documenting outcomes from the children that failed the vision screening. Effective strategies to improve upon low follow-up rates could be further strengthening communication efforts to unaware parents, highlighting the critical importance of following up in a timely manner, and facilitating support to families to find and reach the appropriate eye care specialist [10].

The W.H.E.E.L.S. program's most recent screening term (screening year 4) refractive outcomes determined by the Plusoptix S12 Hand-held Photoscreener (Plusoptix Inc., Nuremburg, Germany) in the children that passed the screening were compared to those that failed; see Table 3. This screening year represented 2945 children that were screened, and 818, or 28% of the children that failed the screening, were recommended for referral; see Table 2. The children represented in the failed group on average had more diopters in sphere and cylinder in both eyes when compared with the children in the passed group. The screening year 4 pupil and gaze asymmetry characteristics in the children that passed the screening compared to those that failed can be seen in Table 4. The pupil size for both eyes and the pupillary distance were similar for both the passed group and the failed group. The gaze asymmetry measurement was slightly higher in the failed group on average when compared with the children in the passed group. Significant refractive errors outside the normal parameters were more frequently the cause for a failed exam compared with pupil or gaze asymmetry errors.

## 4. Conclusion

Reported are the initial vision screening results for 12,402 preschoolers screened by the W.H.E.E.L.S. program using the Plusoptix Photoscreening technology that has been collected since the program was initiated in 2010. The program has identified a high number of preschoolers with significant amblyopic risk factors each screening year that were previously undetected. Identifying the risk factors for amblyopia, the leading cause of impairment in vision in children, includes using referral parameters within the photoscreening device to recognize significant anisometropia, astigmatism, myopia, hyperopia, anisocoria, or gaze asymmetry abnormalities [11–13]. Vision screening regulations differ in each state in the United States since there is not currently a federally mandated uniform vision screening law. The W.H.E.E.L.S. program was developed as a Kindergarten readiness specific initiative as there was no standardized method for screening preschool children in place in Virginia. In general, most routine vision screening evaluations are performed at the child's well-child check-up visits or by a licensed healthcare professional at the child's school, although there are perceived barriers that may limit achieving an optimal referral catch rate [14, 15]. Photoscreening provides an objective and reliable method to identify young children with visual characteristics that are outside the normal parameters [16, 17]. Although follow-up reporting outcomes from children who fail a vision screening examination may be challenging to achieve at high rates, having a mobile vision screening program that uses photoscreening technology in screenings targeting children prior to school entry is an efficient, cost-effective, and noninvasive way to assist in identifying a significant number of children that need to be referred to an eye care specialist [13, 18].

## Figures and Tables

**Table 1:** Vision screening referral parameters defined.

Referral diagnosis	Referral parameter
Anisometropia	Difference of 1.00 D or greater in spherical equivalents
Astigmatism	Cylinder of 1.50 D or greater
Myopia	Spherical equivalent of −0.75 D or greater
Hyperopia	Spherical equivalent of 2.50 D or greater
Anisocoria	1 mm or greater difference in pupil size
Gaze Asymmetry	10-degree difference or greater

**Table 2:** The number of children scheduled for vision screening by the W.H.E.E.L.S. program, the number of actual vision screenings performed, and the number of children who met the referral criteria.

Screening year	Number of scheduled	Number of screened	Number of referrals
Year 1	3387	2796	639
Year 2	3926	3304	787
Year 3	4113	3357	774
Year 4	3649	2945	818

Total	15,075	12,402	3,018

**Table 3:** Screening year 4 mean and standard deviation refractive characteristics of passed versus failed groups.

	Sphere [dpt] OD	Cylinder [dpt] OD	Axis [°] OD	Sphere [dpt] OS	Cylinder [dpt] OS	Axis [°] OS
Passed	0.95 (STDEV 0.57)	−0.45 (STDEV 0.29)	80.00 (STDEV 53.32)	0.94 (STDEV 0.57)	−0.45 (STDEV 0.29)	85.75 (STDEV 52.63)
Failed	2.07 (STDEV 1.28)	−1.55 (STDEV 1.07)	66.10 (STDEV 60.63)	2.08 (STDEV 1.27)	−1.52 (STDEV 1.01)	101.41 (STDEV 63.59)

Total	1.09 (STDEV 0.79)	−0.59 (STDEV 0.59)	78.24 (STDEV 54.47)	1.09 (STDEV 0.79)	−0.58 (STDEV 0.57)	87.73 (STDEV 54.36)

**Table 4:** Screening year 4 mean and standard deviation pupil and gaze asymmetry characteristics of passed versus failed groups.

	Pupil OD [mm]	Pupil OS [mm]	Gaze asymmetry [°]	Pupillary distance [mm]
Passed	5.85 (STDEV 0.86)	5.85 (STDEV 0.87)	2.04 (STDEV 0.99)	53.15 (STDEV 3.43)
Failed	5.93 (STDEV 0.91)	5.93 (STDEV 0.89)	2.48 (STDEV 1.78)	53.74 (STDEV 3.71)

Total	5.86 (STDEV 0.86)	5.86 (STDEV 0.87)	2.10 (STDEV 1.13)	53.22 (STDEV 3.47)
